# Inaccuracies of deterministic finite-element models of human middle ear revealed by stochastic modelling

**DOI:** 10.1038/s41598-023-34018-w

**Published:** 2023-05-05

**Authors:** Arash Ebrahimian, Hossein Mohammadi, John J. Rosowski, Jeffrey Tao Cheng, Nima Maftoon

**Affiliations:** 1 Department of Systems Design Engineering, University of Waterloo, Waterloo, ON Canada; 2 Centre for Bioengineering and Biotechnology, University of Waterloo, Waterloo, ON Canada; 3 Eaton-Peabody Laboratories, Massachusetts Eye and Ear, Boston, MA 02114 USA; 4 Department of Otolaryngology-Head and Neck Surgery, Harvard Medical School, Boston, MA 02114 USA

**Keywords:** Biomedical engineering, Statistical methods, Auditory system, Computational biophysics

## Abstract

For over 40 years, finite-element models of the mechanics of the middle ear have been mostly deterministic in nature. Deterministic models do not take into account the effects of inter-individual variabilities on middle-ear parameters. We present a stochastic finite-element model of the human middle ear that uses variability in the model parameters to investigate the uncertainty in the model outputs (umbo, stapes, and tympanic-membrane displacements). We demonstrate: (1) uncertainties in the model parameters can be magnified by more than three times in the umbo and stapes footplate responses at frequencies above 2 kHz; (2) middle-ear models are biased and they distort the output distributions; and (3) with increased frequency, the highly-uncertain regions spatially spread out on the tympanic membrane surface. Our results assert that we should be mindful when using deterministic finite-element middle-ear models for critical tasks such as novel device developments and diagnosis.

## Introduction

The middle ear plays a vital role in our hearing process by converting acoustic energy from the environment to mechanical vibrations and conducting them to the inner ear. Many studies have used different methods to model middle-ear mechanics in order to improve our fundamental understanding of the hearing process^1–7^. Middle-ear models can be helpful for other purposes such as predicting the hearing loss from middle ear pathologies and injuries, simulating surgeries, and developing and advancing diagnostic and treatment methods.

Different approaches for modelling middle-ear mechanics were reviewed elsewhere^8,9^. The finite-element (FE) method is a powerful continuum-mechanics-based method that has been extensively used to model middle-ear mechanics starting with the pioneering work of Funnell and Laszlo^10^. The FE method can deal with complex geometries and different material properties and boundary conditions.

In order to obtain reliable results from an FE model, realistic mechanical properties of different structures of the middle ear should be known. Several studies attempted to identify the mechanical properties of some of the structures in the middle ear^11–17^. For instance, laser Doppler vibrometry, stroboscopic holography, and FE modelling were used to estimate the viscoelastic properties of the human tympanic membrane (TM)^11^. Also, indentation measurements and inverse FE method were used to estimate the quasi-static Young’s modulus of the human TM^12^. Recently, a Bayesian inverse method was proposed that can be used to find the material properties of thin structures including the TM^13^ using vibration measurements with holographic methods^18–21^. A review of the material characterization of the TM can be found elsewhere^22^. Additionally, several studies have employed sensitivity analysis methods to identify the most influential parameters in the middle ear. In most of these studies, local sensitivity analysis was performed by varying the value of one model parameter while keeping all other model parameters fixed at their baseline values. These one-at-a-time sensitivity analyses can study the local effects of perturbation of parameters around one point in the N-dimensional parameter space (N being the number of model parameters) but they cannot investigate the entire parameter space. Maftoon et al. performed a local sensitivity analysis of the FE model of gerbil middle ear^6^. Motallebzadeh et al. used both local and Morris sensitivity analysis methods to study the effects of variations of parameters of a human newborn FE model^23^. O’Conner et al. studied the effects of varying material properties of the human TM on the sound conduction in the middle ear^24^. In general, the focus of these sensitivity analyses was to find the importance of each of the model parameters rather than studying the impacts of natural stochastic variabilities of the model parameters on the variabilities of the motions in the middle ear.

The mechanical/geometrical properties of middle-ear structures have intrinsic inter-individual variability that can be taken into account with stochastic uncertainties^25^. Indeed, differences observed in physiological data from different individuals (e.g., the normative study by Whittemore et al.^26^) can be attributed to the probabilistic distribution of the morphological features and mechanical properties of the middle-ear structures among individuals. Since the first FE model of the middle ear in 1978^10^, most FE middle-ear models in the literature were deterministic and few systematically considered the effects of these uncertainties in the model parameters on its predictions. In deterministic FE models, the value of all model parameters are predetermined (either from experimental measurements or model sensitivity analyses) and fixed, and stochastic variations of parameters are not integrated in these models. In the present work, we developed a stochastic FE model of the human middle ear to investigate the effects of different levels of natural variability in the model parameters on its outputs.

## Materials and methods

### Geometry, model components, and mesh

The 3D geometry was created based on the segmentation of µCT image datasets of a 73-year-old male cadaver temporal bone that included 1024 × 1012 × 1014 cubic voxels with a voxel size of 18.0828 µm. The scan was done with Xradia MicroXCT-200 at 90 kV and 8W. The segmentation process and meshing were done in 3D Slicer (www.slicer.org) software (version 4.11.20200930)^27^. After finalizing segmenting all parts, we used the Segment Mesher toolbox of Slicer for creating the mesh. We used Cleaver2 (www.sci.utah.edu/cibc-software/cleaver.html) meshing library for creating a conformal volumetric mesh for all parts of the model. A sizing field was created manually and was modified several times in order to create the desired mesh that consists of coarse elements in the regions where no considerable deformations are expected (i.e., bones) and fine elements in other regions. For the TM only, the generated volumetric mesh was converted to a surface mesh and only the medial section of the mesh (which is connected to manubrium) was used in the FE model. Thus, our model had volumetric mesh for all components in the middle ear except the TM for which we had surface mesh. The details about the number of elements will be provided later in this section.

**Figure 1:**
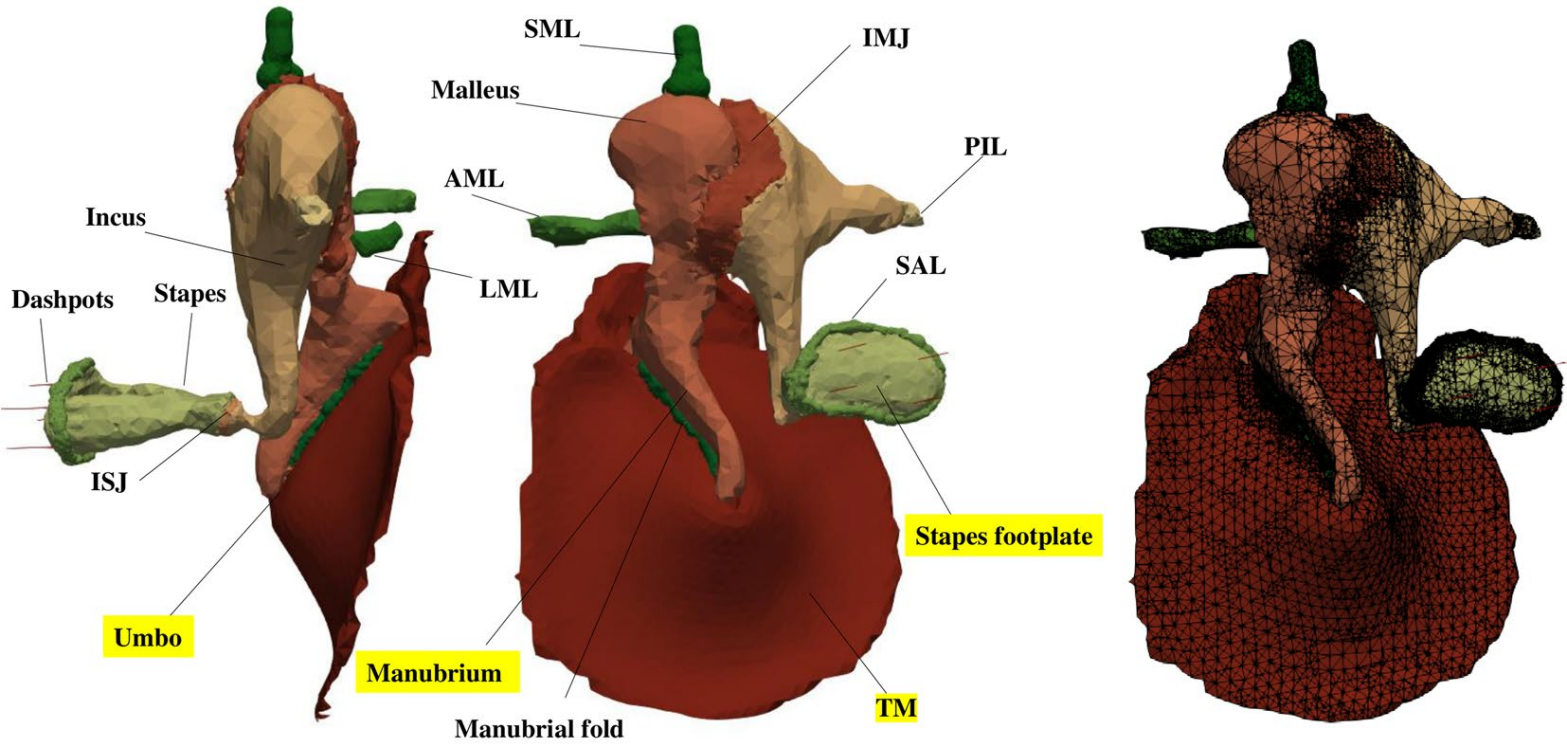
3D model of the middle ear. Constructed geometry from two different viewing angles (left and center) and mesh of the human middle-ear model (right). This model was used in our stochastic FE analysis and the motions of the highlighted structures are discussed in this study.

The model included the TM, ossicles, anterior mallear ligament (AML), lateral mallear ligament (LML), incudostapedial and incudomallear joints (IMJ and ISJ), superior malleolar ligament (SML), stapedial annular ligament (SAL), posterior incudal ligament (PIL), and manubrial fold. The tensor tympani tendon and stapedial tendon were not included in the model as these tendons tend to have functional consequences in live ears, while the experimental data that were used for validations in this study are from cadaveric temporal bones. Also, our model does not include the middle ear cavity. The geometry and mesh of the middle-ear model are shown in Fig. 1. In the following, we discuss the motions of the anatomical landmarks highlighted in this figure.

### Deterministic finite-element modelling

The material properties of the baseline model are presented in Table 1. We assumed all materials to be isotropic and elastic^6^. Besides, we considered nearly incompressible material properties (Poisson’s ratio of 0.49) for all soft tissues^6^. For the ossicles, the value of Poisson’s ratio was set to be 0.3^5^. Moreover, the values of the Young’s modulus of the TM and SAL reported in Table 1 were chosen to be in the range of the values reported in the literature^14,22^. We used Rayleigh damping considering stiffness-proportional damping for all structures except for the TM, and manubrial fold^11,28^. In Table 1, ${\alpha }_{1}$ and ${\alpha }_{2}$ are the Rayleigh-damping coefficients of mass and stiffness matrices, respectively.

For modelling the cochlear load, we considered a cochlear impedance of 20 GΩ which is the same value used by Gan et al.^5^. In our model, the surface area of the stapes footplate was measured to be 3.6 mm^2^ which is also in the range reported by Motallebzadeh et al.^23^. From these values, we calculated a viscous damping coefficient of 0.26 Ns/m that we uniformly distributed to four dashpots attached to the stapes footplate and in the direction parallel to the piston-like motion^6^. Using dashpots to model the cochlear load is a common approach in the literature of FE modelling of the middle ear^5,6,28^. The thickness of the TM was assumed to be uniform and it was considered to be 74 μm which is the same as the value used by Gan et al.^5^. The average TM thickness values used in most models in the literature are also close to this value (74 μm)^29^. The references for the values of the Young’s modulus and density of the model components are listed in Table 1. In addition to these a priori material properties, we determined the Rayleigh damping coefficients of all the middle-ear structures by manually adjusting them to closely replicate the experimental measurements of Voss et al.^1^.

**Table 1:** Material properties of the baseline model.

Structure	E (MPa)	Density (kg/m^3^)	Poisson’s ratio	α_1_ (1/s)	α_2_ (s)
TM	12	1200^11^	0.49	700	4 × 10^−6^
Malleus	14000^24^	2390^24^	0.3	0	4 × 10^−7^
Incus	14000^24^	2150^24^	0.3	0	4 × 10^−7^
Stapes	14000^24^	2200^24^	0.3	0	4 × 10^−7^
IMJ	30^24^	1100^24^	0.49	0	13 × 10^−5^
ISJ	30^24^	1100^24^	0.49	0	13 × 10^−5^
SAL	1.4	1200^24^	0.49	0	13 × 10^−5^
PIL	2^24^	1200^24^	0.49	0	13 × 10^−5^
LML	2^24^	1200^24^	0.49	0	13 × 10^−5^
Manubrial fold	1.2^11^	1200^11^	0.49	700	4 × 10^−6^
SML	4.9^5^	1200^30^	0.49	0	13 × 10^−5^
AML	2^24^	1200^24^	0.49	0	13 × 10^−5^

To excite the model, we applied uniform pressure (with the amplitude of 1 Pa) to the entire TM area laterally. The TM annulus was considered to be fully clamped^31^. Also, the ligaments (AML, LML, SML, PIL, and SAL) were considered to be fixed at their distal ends where they normally connect to the wall of the middle-ear cavity. For all simulations except the full-field vibration patterns, we performed a transient dynamic analysis with a uniform pressure step function as the input^6^ and used the implicit Newmark-β scheme^32^. In order to have an unconditionally stable solution, we chose values of β and γ in the Newmark-β scheme to be 0.25 and 0.5, respectively^6^. To obtain the displacement frequency response function, we found the impulse response by differentiating the step response (with respect to time) and then used the fast Fourier transform to construct the frequency response function. The full-field vibration patterns were obtained using harmonic vibration analysis. The Code_Aster (www.code-aster.org) open-source FE code (version 14.4.0) was used for the computations. We modelled the TM using seven-node second-order TRIA7 COQUE_3D shell elements^6^, while all other structures (ossicles, joints, and ligaments) were modelled using ten-node second-order TETRA10 3D solid tetrahedral elements in Code_Aster.

### Mesh dependency

The original mesh of our model consisted of 205,322 tetrahedral elements (TETRA10 3D) for all parts of the model except the TM that was composed of 7778 shell elements (TRIA7 COQUE_3D). In order to check the mesh convergence, we used the Homard^33^ utility of Code_Aster to refine the original mesh. We divided each triangle into four coplanar triangles and as a result, the numbers of tetrahedral elements and triangular elements were increased by factors of eight and four, respectively. It was observed that the refined mesh does not result in substantial changes in the trend and the values of the vibration responses of the umbo and stapes footplate in the frequency range of 100 Hz to 10 kHz. We compared the results at 405 equally spaced frequencies in this frequency range for the displacement amplitude of the umbo and stapes footplate. For the umbo amplitude, the differences between the results obtained from the original and refined meshes were less than 1 dB at 354 frequencies, with a maximum difference of 1.40 dB at 1.24 kHz. Additionally, for the displacement amplitude of the stapes footplate, the difference between the results obtained from the original mesh and refined mesh was less than 1 dB at 321 frequencies, with a maximum difference of 2.11 dB at 1.17 kHz. As a trade-off between the accuracy and computational cost, we chose to perform all calculations with the original mesh.

### Time-step and time-span dependency

We chose the time span of 25 ms (which provides a frequency resolution of 24 Hz). For checking whether the response is affected by increasing this time span, we increased it to 50 ms (which provides a frequency resolution of 12 Hz) and observed less than 0.001 dB difference for both amplitudes of displacement of the umbo and stapes footplate and for all frequencies in the range of 100 Hz to 10 kHz.

We chose the time step of 10 μs (the maximum frequency of 50 kHz) and decreased it to 5 μs (the maximum frequency of 100 kHz). We observed that in the frequency range of 100 Hz to 10 kHz, decreasing the time step caused maximum changes of 0.36 dB (at 7.59 kHz) and 0.44 dB (at 7.59 kHz) in the displacement amplitudes of the umbo and stapes footplate, respectively. Therefore, we used the original time step (10 μs) and time span (25 ms) for all our calculations.

### Uncertainty propagation

We made a baseline conventional deterministic FE model with a priori material parameters from the literature and validated this model against existing experimental data. We then considered the probability distribution of the model parameters, sampled the 32-dimensional parameter space and propagated the uncertainties to the model outputs. We considered having uncertainties in the material properties (Young’s modulus, Poisson’s ratio, and stiffness-proportional damping coefficient) for all of the components of the model as well as in the thickness of the TM and in the cochlear load. All these uncertainties existed at the same time in our simulations.

We quantified uncertainties using two indices: the coefficient of variation (CV):

1 $$\mathrm{CV}=\left|\frac{\text{standard deviation}}{\mathrm{mean}}\right|\times 100,\quad {\text{if mean}}\ne 0$$

and uncertainty amplification (UA) which quantifies the amplification of the uncertainties of the outputs with respect to the uncertainties in the model parameters^34^:

2 $$\mathrm{UA}=\frac{{\mathrm{CV}}_{\mathrm{Output}}}{\frac{1}{n}\sum_{i=1}^{n}{CV}_{Inputs}}$$

where *n* is the number of uncertain model parameters. In our study, we had 32 uncertain model parameters. A UA value of greater than one indicates amplification.

**Figure 2:**
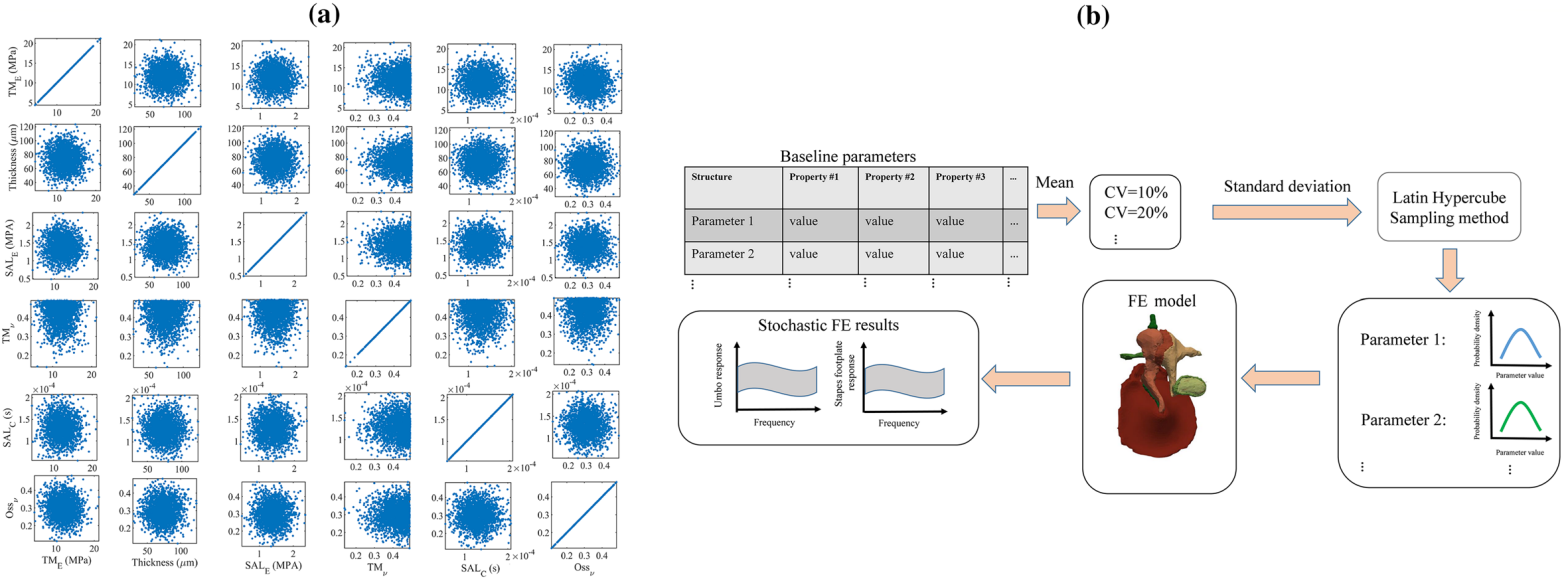
Distribution of some of the uncertain model parameters and workflow of the stochastic FE analysis. Panel (**a**): Some of the 2D views of the 32-dimensional parameter space sampled using the Latin Hypercube method with the coefficient of variation (standard deviation/mean) of 20%. Depending on the nature of the parameter, the sampled space shows normal and half-normal distributions. We used 1992 sets of stochastic model parameters in our stochastic FE analysis. Panel (**b**): Workflow of the stochastic FE analysis process used to study the effects of uncertainty in the middle-ear model. In the first step, we used the values of the baseline model (reported in Table 1) as the mean values of our stochastic sets of model parameters. We then specified the amount of coefficient of variation (standard deviation/mean) of the uncertain parameters. We considered the coefficient of variation to be 10% and 20% in the current work and used the Latin Hypercube Sampling method to create stochastic sets of model parameters with normal and half-normal distributions. The next step was to solve the FE model for all of these stochastic sets of model parameters, and from here, we found the stochastic outputs of the system. We used these stochastic outputs to study the amplification of the uncertainties in the system outputs and determine the output distributions.

The stochastic sets of model parameters were created by the Latin Hypercube Sampling method for each scenario (each combination of random parameters sampled from the 32-dimensional parameter space) with the mean values equal to values for the baseline model (Table 1 for material properties, TM thickness of 74 μm, and cochlear load viscous damping coefficient of 0.26 Ns/m) and CV of 10% and 20%. The ranges of variation of each model parameter for both CV values are reported in Supplementary Table S1. We used UQLab^35^ (version 1.3.0) for creating stochastic sets of model parameters. In the absence of reported probability distributions for most parameters of the model, we assumed normal distribution, which is advocated to be a suitable choice for many biological variables^36^. However, because the value of the Poisson’s ratio was set to 0.49 for soft tissues in the baseline model, we considered a half-normal distribution for soft tissues with the mean of 0.49 that only resulted in values less than or equal to the mean. Also, in order to reduce the number of parameters, we considered all three ossicles to have the same Young’s modulus, Poisson’s ratio, and damping in our stochastic modelling. Visualization of the sampled 32-dimensional parameter space is not directly possible but Fig. 2a shows the distribution of parameters on the planes of some pairs of parameters of the space with CV of 20%. The planes in this figure show normal and half-normal distributions as described above.

The uncertainty in the model parameter space was propagated to the output by performing FE analysis for each set of the stochastic model parameters described above. We evaluated the FE model with 1992 parameter scenarios to cover the 32-dimensional parameter space. All of the FE calculations were performed on the Niagara cluster of Digital Research Alliance of Canada (www.alliancecan.ca) with Intel Skylake (2.4 GHz, AVX512) processors running under the CentOs 7 distribution of Linux. Each single simulation of the model with the original mesh and original time step and time span (“Results”) was performed on one core using 18 GB memory. Each node of the cluster has 40 cores and 188 GiB of memory, allowing 10 parallel simulations on one node and a total number of 100 model evaluations on one node took about 14 h to complete.

After propagating the uncertainties to the outputs, statistical analyses were done on the outputs. For the output phases, we implemented circular statistics to find values of the mean, standard deviation, skewness, and kurtosis using the Circular Statistics Toolbox of Matlab^37^. Using circular statistics, we consider the circular nature of all of these parameters, which is important for phase values. For instance, the circular standard deviation is analogous to linear standard deviation but it considers the cyclic nature of phase values when evaluating their variabilities. More details about these parameters can be found in the documentation of the Circular Statistics Toolbox^37^. To calculate the skewness and kurtosis of the output phase, we employed the methods described by Pewsey^38^ and Fisher^39^, respectively.

Figure 2b provides a summary of the workflow of the stochastic FE analysis that we used in the current work.

We submitted 2000 simulation scenarios on the cluster from which eight faced software issues and we could evaluate the FE model with 1992 parameter scenarios to cover the 32-dimensional parameter space.

## Results

### Middle-ear model can magnify parameter uncertainties up to more than three times in the output

**Figure 3:**
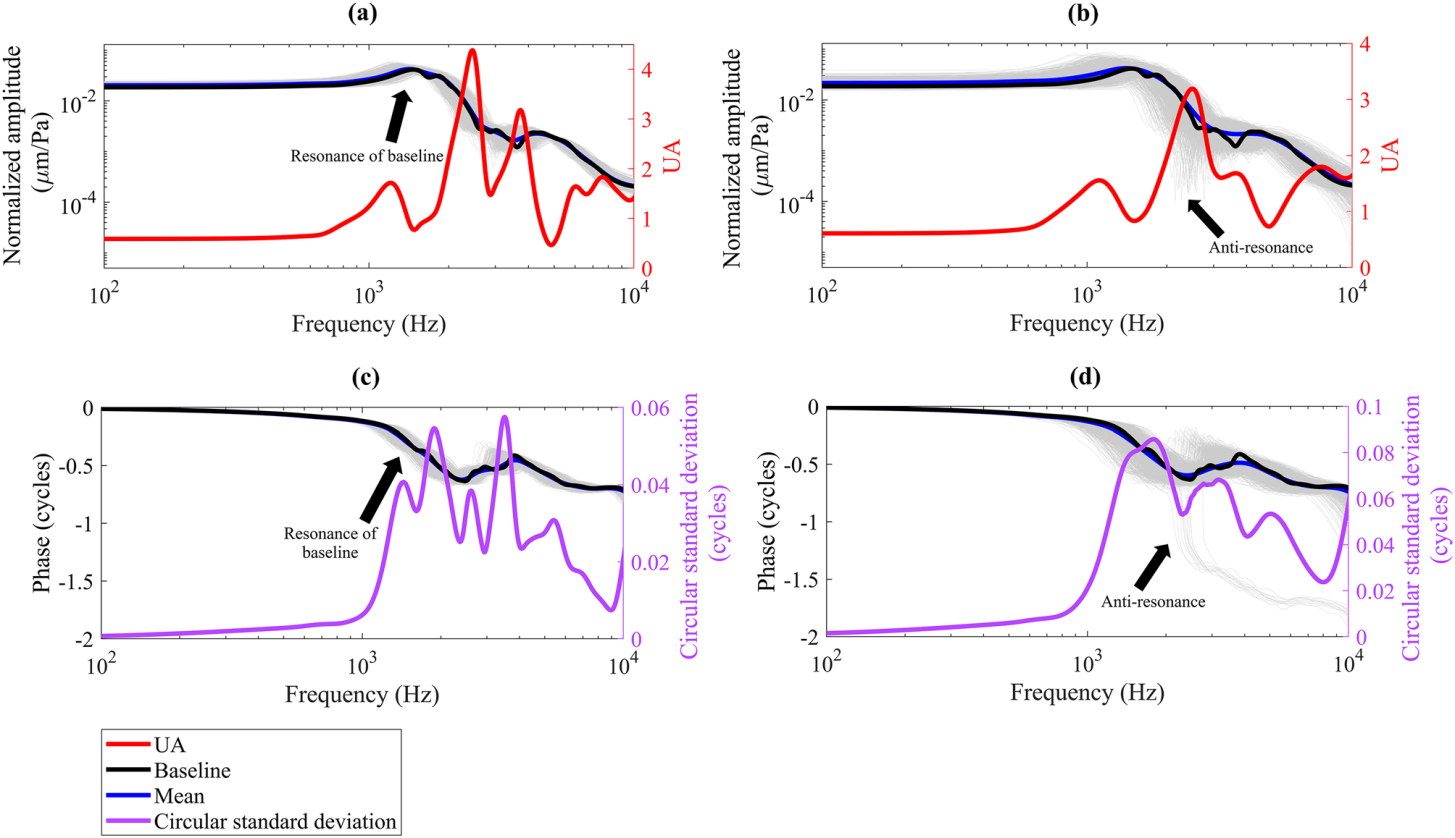
Stochastic frequency response function of the umbo displacement. Amplitude and phase of the umbo (normalized with respect to excitation pressure) are presented in this figure. The motion of the umbo is reported in the direction normal to the manubrium at the umbo. The results of individual simulations (n = 1992) are plotted with gray thin lines and because they are all close to each other, the whole response region looks like a gray shaded area. Panels (**a**) and (**c**): The CV of all uncertain model parameters was set to be 10%. The UA (Equation (2)) is about 0.6 for the umbo displacement amplitude below the middle-ear resonance frequency (at ~ 1.5 kHz). The maximum UA of about 4.38 happens near 2.5 kHz for the umbo displacement amplitude. For the phase, the values of circular standard deviation are close to zero at low frequencies and the maximum circular standard deviation happens near frequencies of 1.9 kHz and 3.5 kHz. Panels (**b**) and (**d**): The CV of all uncertain model parameters was set to be 20%. The UA (Equation (2)) is about 0.6 for the umbo displacement amplitude at frequencies below the middle-ear resonance frequency (at ~ 1.5 kHz). For the amplitude, the maximum UA (3.19) happens near about 2.5 kHz and for the phase, the maximum circular standard deviation (0.09 cycles) happens at about 1.8 kHz.

Figure 3a and c, respectively, present the stochastic normalized amplitude and phase of the umbo displacement for model parameters with the CV of 10%. The middle-ear resonance frequency, identified by the peak in the umbo amplitude response, was about 1.5 kHz in the deterministic baseline response and had a UA of about 0.77 in the stochastic model. At low frequencies (below the middle-ear resonance frequency), the uncertainty of the umbo displacement amplitude was not amplified (UA of about 0.6). Also, the value of the circular standard deviation of the phase remained close to zero at low frequencies. Above the middle-ear resonance frequency, the uncertainties were amplified in the umbo displacement output with the highest UA of 4.38 near 2.5 kHz for the amplitude and maximum values of the circular standard deviation in the phase (0.06 cycles) happened near frequencies of 1.9 kHz and 3.5 kHz.

Increasing the uncertainty in the model parameters to the CV of 20% (Fig. 3b,d) did not lead to uncertainty amplification at low frequencies: the UA of the amplitude remained about 0.6 and the circular standard deviation values of the phase remained close to zero. Also, the UA of the middle-ear resonance frequency experienced a negligible increase (0.83 vs. 0.77). At frequencies higher than the middle-ear resonance, the increased uncertainty in the model parameters caused more dispersed umbo displacement responses with an average CV of about 32% for amplitude (was about 17% when CV of inputs were 10%) and an average standard deviation of 0.05 cycles in phase (was 0.02 cycles for CV of 10%). Near 1.8 kHz, the circular standard deviation of the phase shows its maximum (0.09 cycles). Also, the greatest UA peak (3.19) of the umbo displacement amplitude was in the vicinity of 2.5 kHz. In this frequency neighborhood, for some sets of model parameters, an anti-resonance occurred as can be seen in Fig. 3b and d. These anti-resonances did not exist in Fig. 3a and c where the CV of uncertain model parameters was 10%.

**Figure 4:**
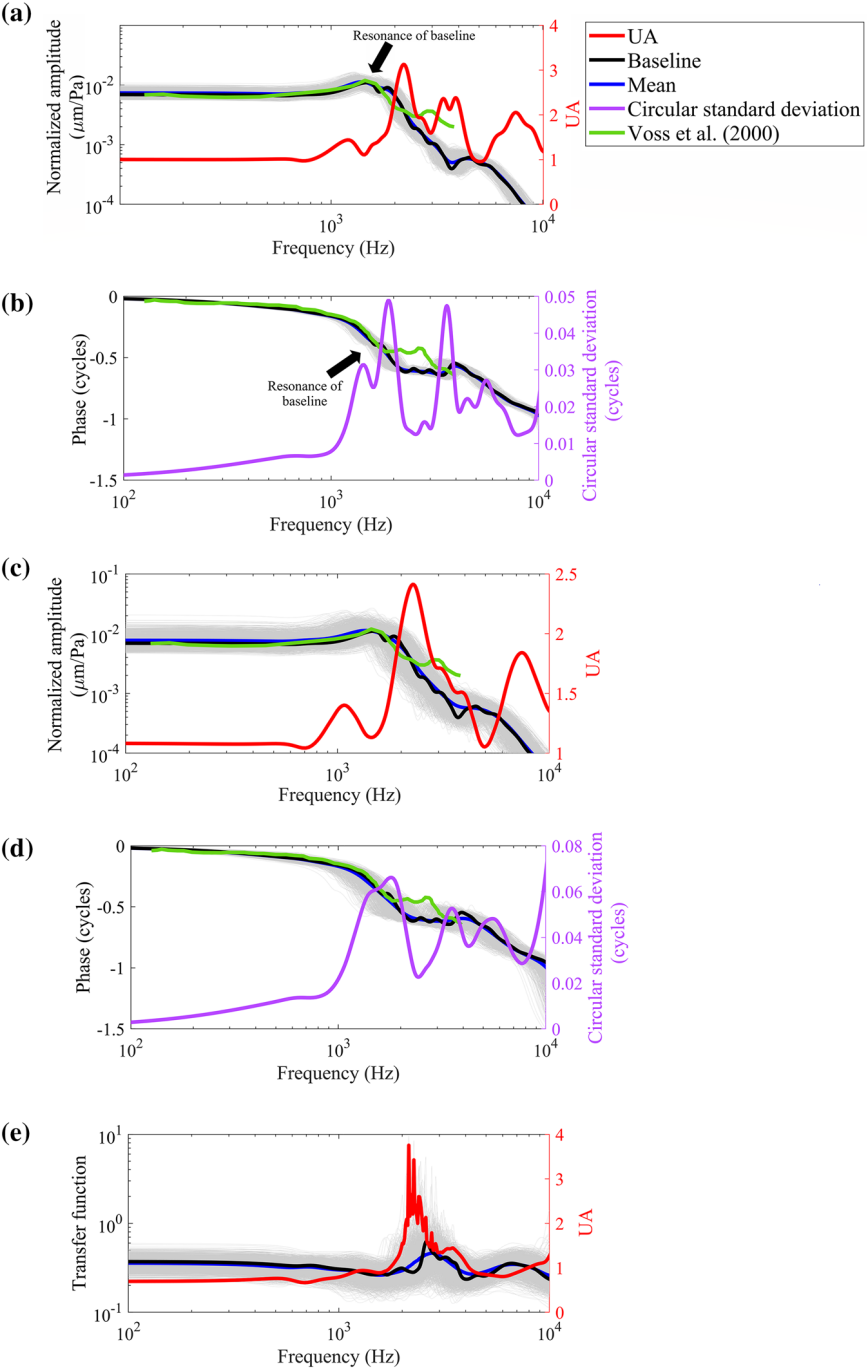
Stochastic frequency response function of the stapes footplate displacement and the stochastic middle-ear transfer function. The results of individual simulations are plotted with gray thin lines and because they are all close to each other, the whole response region looks like a gray shaded area. The stapes footplate motion is reported in the piston-like direction. Also, the green line presents the experimental results of Voss et al.^1^ (Bone 25) converted to displacement and corrected for the viewing angle of 35°; the mean of their reported viewing angle range of 20°–50° degrees. Panels (**a**) and (**b**): Amplitude and phase of the stapes footplate (normalized with respect to excitation pressure) with the CV of of 10% for all uncertain model parameters. At frequencies below the middle-ear resonance frequency (at ~ 1.5 kHz), the UA (Equation (2)) is about 1 for the stapes displacement amplitude. The maximum UA (3.12) happens near 2.2 kHz for amplitude and the maximum circular standard deviation (0.05 cycles) happens near the frequencies of 1.9 kHz and 3.6 kHz for the phase. Panels (**c**) and (**d**): Amplitude and phase of the stapes footplate (normalized with respect to excitation pressure) with the CV of 20% for all uncertain model parameters. The UA (Equation (2)) is about 1.1 for amplitude at frequencies below the middle-ear resonance frequency (at ~ 1.5 kHz). The maximum UA (2.41) happens near 2.3 kHz for the stapes displacement amplitude and the greatest circular standard deviation happens (0.07 cycles) near frequencies of 1.8 kHz and 10 kHz for the phase. Panel (**e**): Stochastic middle-ear transfer function calculated using the stochastic model with CV of 20% in model parameters (Fig. 3b and Fig. 4c). The maximum UA of the lever ratio (3.75) happens at about 2.1 kHz.

The stapes displacement describes the output of the complete middle ear. Figure 4a and b show the frequency response of the stapes footplate with the CV of 10% for all uncertain model parameters. Similar to the umbo response, at low frequencies (below the middle-ear resonance at 1.5 kHz), uncertainty amplification did not occur: the UA was about 1 for the amplitude and the values of the circular standard deviation were close to zero. For frequencies above the middle-ear resonance frequency, the model amplified the uncertainty in the stapes response (average amplitude UA: 1.72 and average phase circular standard deviation: 0.02 cycles). The maximum UA of 3.12 happened at about 2.2 kHz for the stapes displacement amplitude. Also, for the phase, the maximum circular standard deviation of 0.05 cycles happened near frequencies of 1.9 and 3.6 kHz.

Also, the open-cavity stapes footplate velocity data of Voss et al. are presented in Fig. 4. We compared the stapes displacement amplitude of the baseline model with these experimental results and found that the maximum difference was less than 1.6 dB for all frequencies below the middle-ear resonance frequency (1.5 kHz). Also, as Fig. 4 displays, the middle-ear resonance frequency and the maximum stapes footplate displacement amplitude obtained from the model match well with the experimental data.

Figure 4c and d present the frequency response of the stapes footplate for the CV of 20% for all uncertain model parameters. At low frequencies, the UA was about 1.10 for the amplitude and the circular standard deviation values of the phase were close to zero. At 2.3 kHz, the amplitude response was most uncertain and the uncertainty in the model parameters was magnified by a factor of 2.41 while the circular standard deviation of the phase response drastically decreased at this frequency to 0.02 cycles (from 0.07 cycles at 1.8 kHz) and went up to 0.07 again near the frequency of 10 kHz. Here the anti-resonances, observed in the umbo response (Fig. 3b,d), are not present. Compared to panels (a) and (b) of Fig. 4, the increased uncertainty (CV of 20%) in the model parameters, caused an approximately similar average amplitude UA (1.58 vs. 1.72) and a higher average phase standard deviation (0.04 cycles vs. 0.02 cycles) and the stapes response was in a better agreement with the experimental results. A comparison of the stochastic model results of the stapes footplate (with CV of 20%) with several experimental measurement results in the literature is also provided in Supplementary Fig. S1.

### At low frequencies, middle-ear models attenuate uncertainties and approximate a robust simple lever

Figure 4e presents the stochastic ossicular transfer function of the model with the CV of 20% for the uncertain model parameters. This ossicular transfer function is defined as the ratio of the stapes footplate displacement amplitude (in the piston-like direction) to the umbo displacement amplitude (in the direction normal to the manubrium at the umbo). At low frequencies (below the middle-ear resonance), the transfer function can be thought as the lever ratio (at low frequencies, the mean value of the lever ratio is less than 0.4 and the UA of the lever ratio is about 0.7). However, UA increases up to a maximum of about 3.75 near 2.1 kHz. The uncertainties in the model parameters make the transfer function highly uncertain at most frequencies between 2 and 4 kHz. At the frequencies below 1 kHz, the model robustly attenuates uncertainties and portrays the middle ear as a highly certain simple lever. The same trend of attenuating uncertainty at low frequencies is evident in all results of Figs. 3 and 4 as discussed earlier.

### The middle-ear model is biased

**Figure 5:**
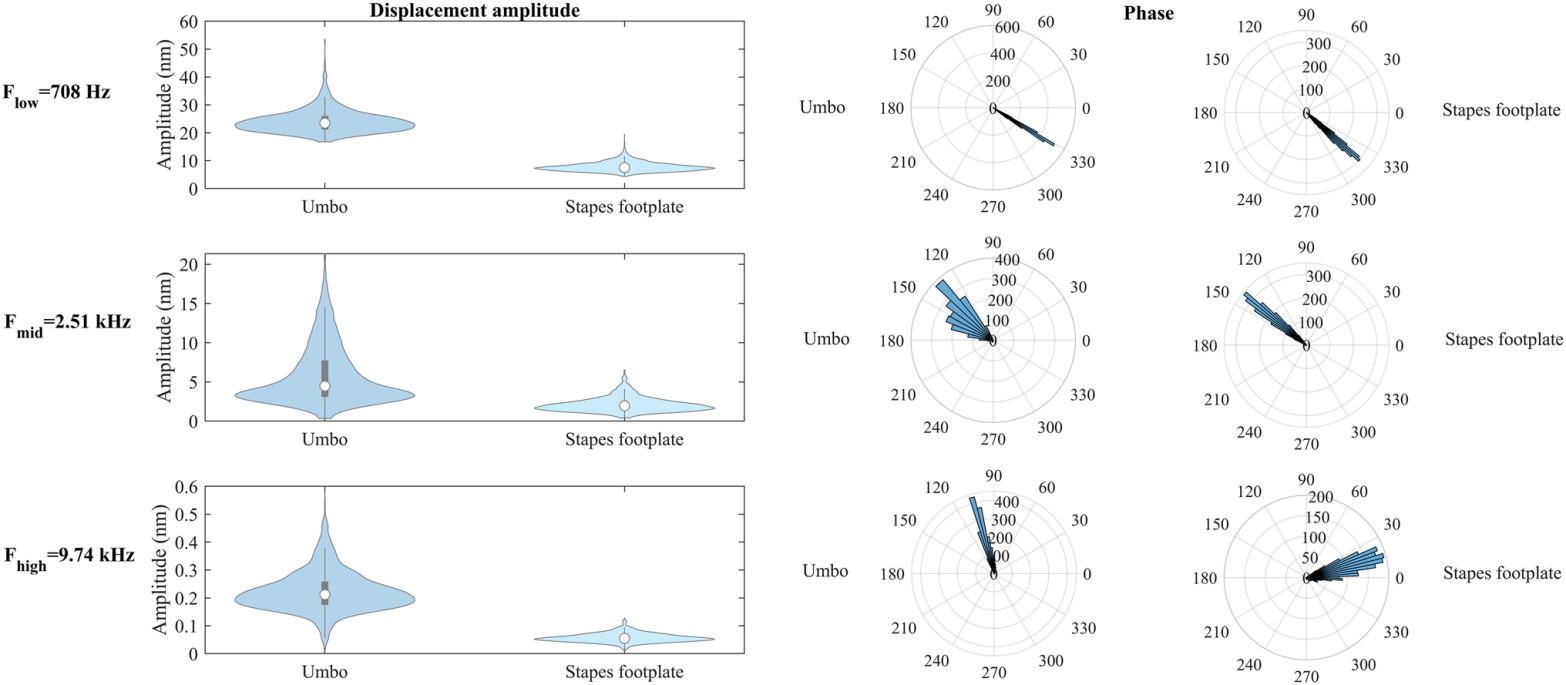
Violin plots and circular histograms for the amplitude and phase of the umbo and stapes footplate. The results are presented at frequencies F_low_, F_mid_, and F_high_. The CV of uncertain model parameters was set to be 20%. The horizontal axes in the violin plots show kernel density estimations for each distribution. The values of skewness and kurtosis of the distributions shown in this figure are presented in Table 2. We used Kuiper’s normality test to check whether the distributions shown in this figure are normal (von Mises for circular distributions^41^). Based on Kuiper test results presented in “Results”, the null hypothesis was rejected at 5% significance level except for the phase of the stapes footplate at F_low_.

To study the probability distributions of stochastic responses at low, mid, and high frequencies, we focused on three frequencies (*F*_*low*_ = 708 Hz, *F*_*mid*_ = 2.51 kHz, and *F*_*high*_ = 9.74 kHz). Figure 5 presents violin plots^40^ of the amplitude and circular histograms of the phase of both umbo and stapes footplate displacement at these frequencies (for CV of 20% for uncertain model parameters). The horizontal axes in the violin plots show kernel density estimations for each distribution. We used Kuiper’s normality test to check whether the distributions shown in Fig. 5 are normal (von Mises for circular distributions^41^). We found that for all outputs, the null hypothesis was rejected at 5% significance level except for the phase of the stapes footplate at *F*_*low*_. This means that although most of the uncertain model parameters were considered to have normal distributions, the output distributions may be distorted with non-normal distributions.

The values of skewness (a measure of symmetry of a specific distribution) and kurtosis (a measure of the tail weight of a specific distribution with respect to that of a normal distribution) reported in Table 2 also confirm that most of the distributions presented in Fig. 5 are non-normal as their skewness and kurtosis values are far from the respective values for the normal distribution (skewness of zero and kurtosis of three for the amplitude^42,43^ and skewness and kurtosis of zero for the phase^37^). For the amplitude of the umbo displacement, the absolute value of skewness is greater than 0.90 at all three frequencies with a maximum absolute value of 1.59 at *F*_*low*_. Also, the values of kurtosis for the amplitude of the umbo displacement are greater than 4.12 at all three frequencies with the maximum value of 7.88 at *F*_*low*_. For the phase of the umbo displacement, the value of skewness is very close to zero at all three frequencies. Additionally, the values of kurtosis of the phase of the umbo displacement are about 1.94, 12.70, and 19.72 at frequencies *F*_*low*_, *F*_*mid*_*, and F*_*high*_, respectively. These findings are consistent with the results of the normality test above.

**Table 2:** Values of skewness and kurtosis for the output distributions shown in Fig. 5.

Frequency	F_low_ (708 Hz)		F_mid_ (2.51 kHz)		F_high_ (9.74 kHz)	
Statistical information	Skewness	Kurtosis	Skewness	Kurtosis	Skewness	Kurtosis
Umbo amplitude	1.59	7.88	1.25	4.12	0.90	4.63
Umbo phase	0.00	1.94	− 0.06	12.70	0.05	19.72
Stapes footplate amplitude	1.17	5.80	1.27	5.15	0.69	4.22
Stapes footplate phase	0.00	0.88	− 0.01	17.48	0.07	14.27

For the displacement amplitude of the stapes footplate, the value of skewness is greater than 1.17 at all three frequencies with a maximum value of 1.27 at *F*_*mid*_. Furthermore, the values of kurtosis for the amplitude of stapes displacement are greater than 4.22 at all three frequencies with a maximum of 5.80 at *F*_*low*_. Table 2 also shows that, like the umbo response, the values of skewness for the phase of the displacement of the stapes footplate are very close to zero at all three frequencies. Besides, the values of kurtosis for the phase of the displacement of the stapes footplate are greater than 0.88 at all three frequencies with the maximum value of 17.48 at *F*_*mid*_.

### Uncertainties in the vibration patterns of the TM are spread in space with increased frequency

**Figure 6:**
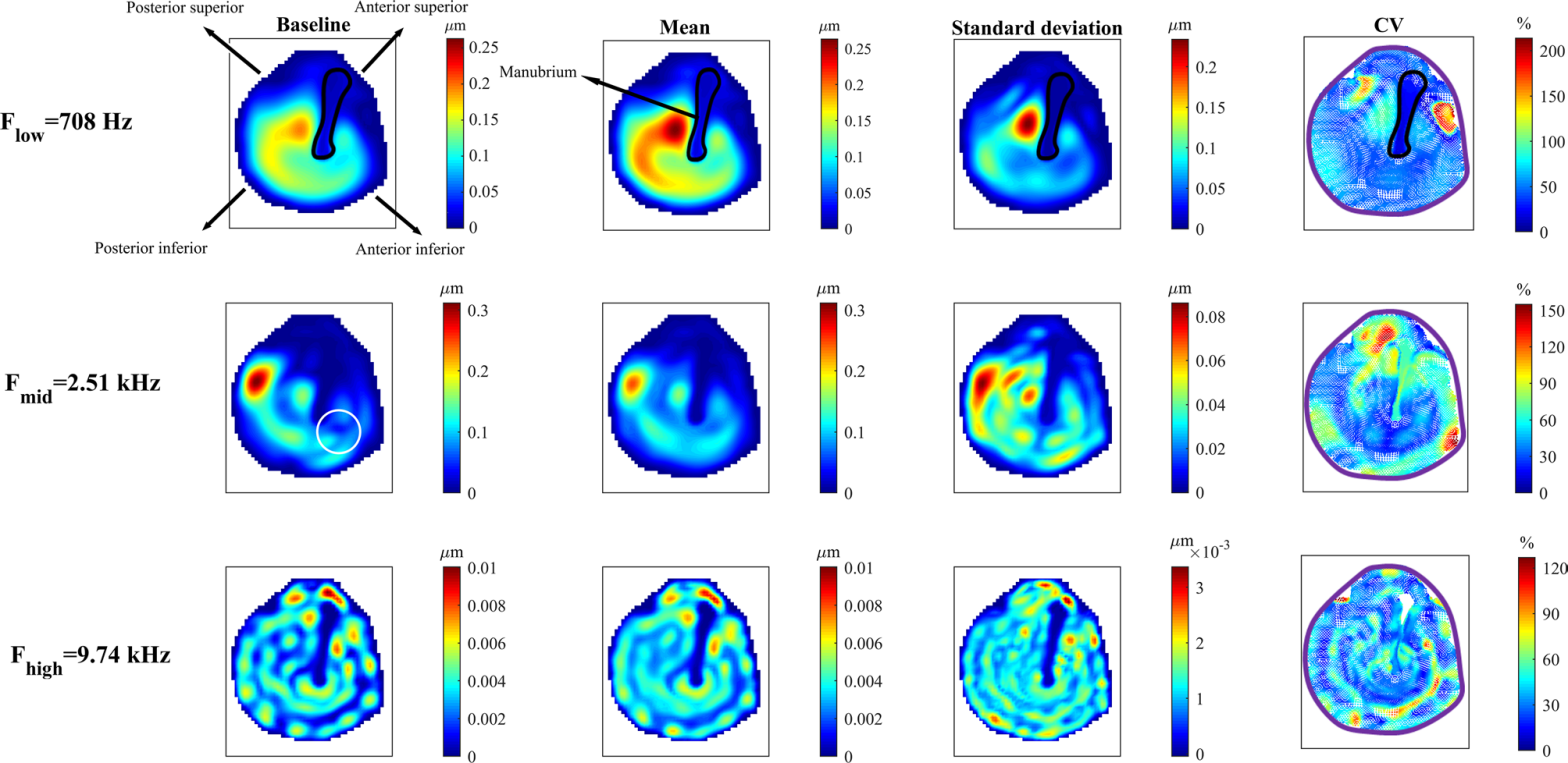
Stochastic full-field vibration patterns of the TM. Vibration patterns of the baseline model and the spatial distribution of the mean and standard deviation of the displacement amplitude of the tympanic membrane at three frequencies F_low_, F_mid_, and F_high_ are presented in the three left columns. The vibration patterns are reported in the direction normal to the manubrium at the umbo. Comparing the mean and baseline patterns shows that at F_mid_ and F_high_, some high-frequency features are not present in the mean pattern due to the averaging and high variations in the stochastic results. The white circle shows one example of such high-frequency features. The right column shows the distribution of the coefficient of variation, excluding points where the mean values are close to zero. The manubrium is outlined in black. The purple line shows the tympanic annulus.

**Figure 7:**
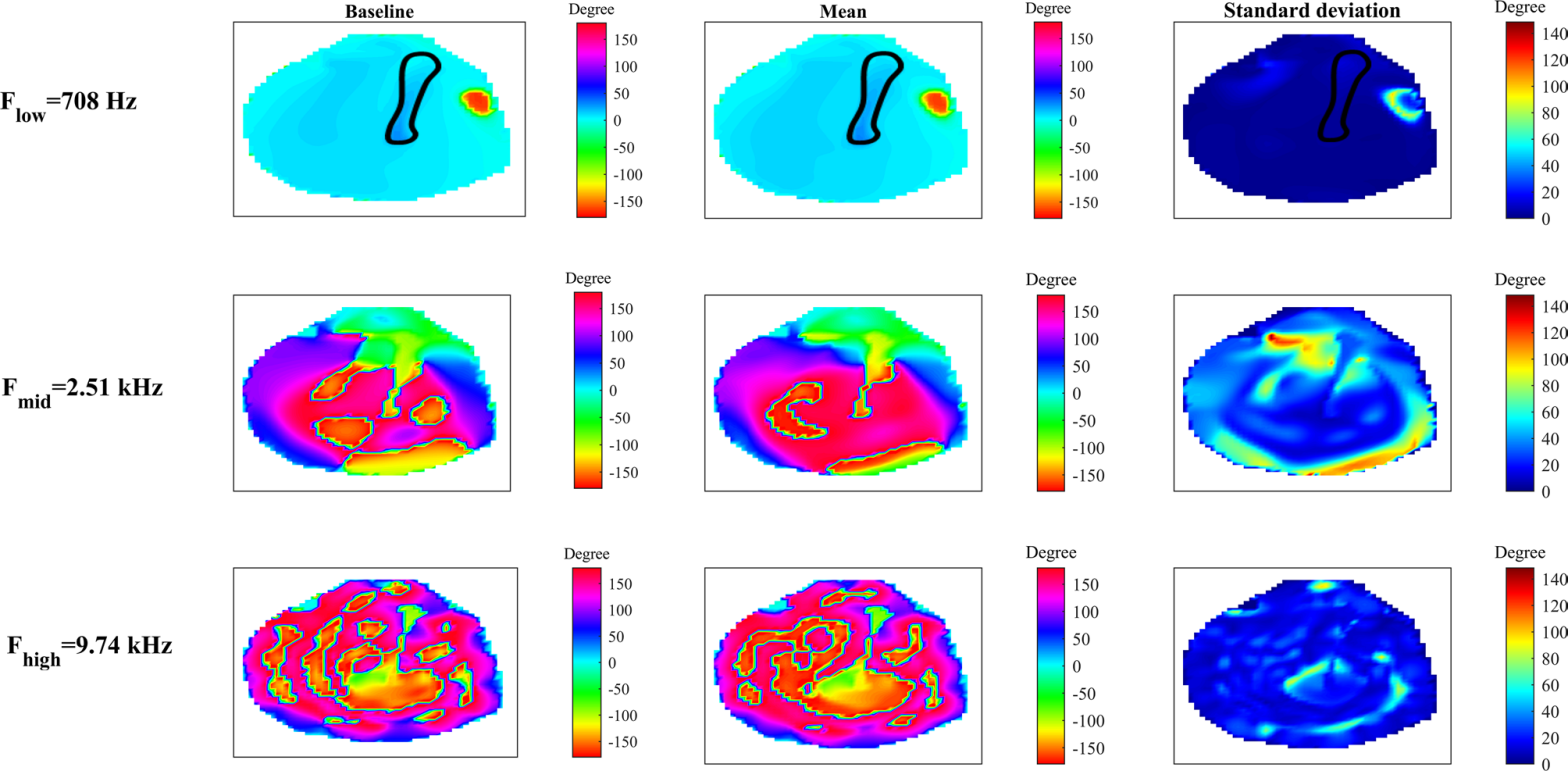
Stochastic full-field vibration phase of the TM. Distribution of the displacement phase of the baseline model and the spatial distribution of circular mean and circular standard deviation of the phase of the tympanic membrane. The results are presented at three frequencies F_low_, F_mid_, and F_high_. The manubrium is outlined in black.

The previous results showed the propagated uncertainties only for the umbo and stapes footplate. To provide a more complete view about the stochastic motions in the model, Fig. 6 presents the vibration amplitude of the TM at the same three frequencies. This figure considers the displacement of the baseline and stochastic FE model with CV of 20% for the model parameters. For the 15,728 nodes of the TM model, we calculated the displacement amplitude and phase in the baseline model as well as their mean and standard deviation in the stochastic model. The results of the amplitude are presented in the three left columns of Fig. 6 and the results for the phase are provided in the Fig. 7. The rightmost column of Fig. 6 presents the values of CV of the displacement amplitude. We should note that only the CV of the nodes of the TM that have mean displacement values of greater than 0.1 nm are plotted in this column. The patterns obtained from the baseline model have similarities to the mean stochastic results shown in the “mean” column. At all three frequencies, the manubrium (outlined in black at *F*_*low*_) has the smallest motion in the baseline and has the smallest values of the mean and standard deviation. At *F*_*low*_, the maximum deformation of about 0.25 µm occurred in the posterior side of the TM (left to the manubrium in Fig. 6), as expected^44,45^. Besides, at this low frequency, the standard deviation plot shows that posterior to the TM, the standard deviation of the motion can be as large as the mean or baseline displacement (also evident from the CV of about 110%). At *F*_*mid*_ and *F*_*high*_, some features in the baseline pattern (one example is marked with the white circle) are not present in the mean pattern because they were smoothed out due to averaging and due to the high variations in the results at these locations. At the middle frequency (*F*_*mid*_), the major area of the TM shows standard deviations of more than 0.02 µm except in the superior region of the TM where standard deviations of down to 0.001 µm dominate the region. Consistently, the CV plot at *F*_*mid*_ shows that regions with high CV (≥ 50%) are apparent in all regions of the TM. Increasing the frequency to *F*_*high*_ leads to nearly even distribution of regions with high standard deviations and high CV on the entire TM, especially in comparison to the vibration patterns at *F*_*low*_. The mean values of CV of the TM nodes (Fig. 6) are about 46%, 56%, and 38% for the *F*_*low*_, *F*_*mid*_, and *F*_*high*_, respectively. This shows that although by increasing the frequency the regions with high CV are spatially spread out on the TM, the mean value of the CV does not follow any specific trends.

The stochastic phases of vibration patterns of the TM are presented in Fig. 7. Compared to the displacement amplitude, the displacement phase has a more evenly distributed standard deviation at *F*_*low*_. Also, at *F*_*low*_, the vibration phase is nearly constant and highly certain over the entire TM except in a small circular region in the TM anterior with concentrated uncertainty and a maximum standard deviation of about 102°. The displacement phase pattern becomes more uncertain as the frequency increases and at *F*_*mid*_ several hot spots with concentrated uncertainty are present in the standard deviation plot. As the frequency increases to *F*_*high*_ the uncertainty is dispersed in the entire TM. The average values of the circular standard deviation at *F*_*mid*_ and *F*_*high*_ (38° and 20°, respectively), are much greater than that at *F*_*low*_ (4°)_*.*_

## Discussions and conclusions

In this study, we presented a stochastic FE model of the human middle ear that instead of generating deterministic vibrations and model outputs, provides a probabilistic description of middle-ear vibrations to account for the natural variabilities in the human middle ear. Current FE models do not have enough accuracy to be considered as acceptable tools for diagnosis and surgical planning^46,47^. This study is a step toward increasing the accuracy and reliability of the predictions of FE models by considering the uncertainty in the model parameters.

To take the variability in individual ears into account, the stochastic model considered uncertainty in the mechanical parameters as well as the thickness of the TM. We demonstrated that the middle-ear model can magnify the uncertainties in the model parameters to more than 300% in the outputs. For both CV values of 10% and 20% of the model parameters, the highest UA happened in the frequency range of 1–5 kHz for the amplitudes of the umbo and stapes displacements. This new finding enhances our existing knowledge^6,48^ about the sensitivity of the middle ear to variability. Uncertainty amplification that we report here was also observed in models of other organs of the body (e.g., left ventricle^49^).

Also, for the model with the CV of 20%, for some sets of model parameters, we saw an additional anti-resonance in umbo displacement at frequencies around 2–3 kHz that does not exist in the baseline model. Based on our results, for the sample sets that lead to these anti-resonances in the umbo response, the values of the thickness or the Young’s modulus of the TM (or both of them for most samples) are small (below mean values). However, the values of other model parameters and their interactions may also have some effects in the presence of these anti-resonances. The presence of anti-resonances for some sets of model parameters is consistent with the presence of similar antiresonances in some ears in experimental observations of populations of middle ears^26,50^. Additionally, the noisy behaviour of the transfer function (Fig. 4e) can be due to these anti-resonances for some sets of model parameters.

All the observations described above have important implications for FE models of the middle ear because in addition to inter-individual variability that makes the model parameters intrinsically uncertain, most material parameters of the models were not and cannot be measured accurately in vivo, at least with the existing methods. If conventional deterministic FE models of the middle ear are to be used for developing new medical devices or for planning therapeutic interventions, the ignored error in the values of material and geometrical parameters (uncertainty) may be amplified as errors in the model results. We observed that the umbo and stapes footplate responses and the middle-ear transfer function are less uncertain at low frequencies. This suggests that if, despite uncertainties, a deterministic model is used, the model predictions at low frequencies are more reliable than those at high frequencies. Moreover, by investigating stochastic vibration patterns of the TM, we observed that as the frequency of excitation increases, the regions with high uncertainty spread out more evenly on the entire TM surface and the vibration pattern becomes less certain. This suggests similar implications to what we discussed for the umbo, stapes footplate and the middle-ear transfer function.

Although the current study reveals the importance of considering using stochastic models to study the mechanics of the middle ear, these models require a significantly higher computational cost in comparison to conventional deterministic FE models. One way to deal with the high computational cost is to develop and train surrogate models^51^ that can be used in lieu of the real FE model for stochastic simulations for some specific output quantities of interest. Future studies should study development and effectiveness of such surrogate models.

One of the limitations of our current stochastic FE model is that we only considered normal and half-normal distributions for the model parameters. We chose these distributions based on the fact that normal distributions are suitable choices for many biological variables^36^. However, the exact distribution of each of the model parameters should be determined by performing further experiments on a large number of human ears.

In the absence of more accurate estimates of the value of the CV of most of the uncertain model parameters, we considered the CV values of 10% and 20%. However, additional experimental measurements should be carried out in order to quantify the CV of each of the model parameters. Recently, Lobato et al. tried to quantify the CV of mechanical parameters of the middle ear^52^ based on the ranges reported in the literature for each value. However, their study does not include all mechanical properties of the middle ear structures. For instance, CV of damping, Poisson’s ratio, and cochlear load is not reported in their work. Additionally, as Lobato et al. also mentioned in their work, even for the parameters reported in their work, the values of CV were calculated from small sample sets and therefore, those values might not be accurate enough. Furthermore, to avoid excessive sophistication of performing this study, we only considered the variability in the TM thickness among all morphological parameters of the middle ear because that was the only morphological variability that would not need creations of new 3D models. Future studies should explore the effects of morphological variability in interaction with material variability.

We have considered some simplifying assumptions to reduce the complexity of our model. For instance, our baseline model does not include the tensor tympani tendon, stapedial tendon, and the middle-ear cavity. The tendons can be activated in living ears but in ex vivo ears they are not active and the stapedial tendon is often removed in temporal-bone preparations. Voss et al. showed that removing the stapedial tendon has small effects on the mechanics of the middle ear ex vivo^1^. Also, the middle-ear cavity is expected to have small effects on the motions of the stapes footplate at many frequencies^1,24^. Moreover, although some studies have modelled the TM as an orthotropic material^22^, in this study we modelled the TM as an isotropic material to reduce the complexity of our model. O’Connor et al. modelled the TM using both orthotropic and isotropic materials and showed that the isotopic material model can also lead to results comparable to the ones from the orthotropic model and close to experimental measurements^24^. All these simplifications may have some effects on the predictions of the present stochastic finite-element analysis and they should be further investigated in follow-up studies.

## Supplementary Information

Supplementary Information.

## Data Availability

The datasets generated during and/or analysed during the current study are available from the corresponding author on reasonable request.
