# Supplementary material for: Inaccuracies of deterministic finite-element models of human middle ear revealed by stochastic modelling
Source: Sci Rep. 2023 May 5;13:7329. doi: 10.1038/s41598-023-34018-w (PMC10163043; doi:10.1038/s41598-023-34018-w)
Supplement: Supplementary file 1 — Supplementary Information. [file 41598_2023_34018_MOESM1_ESM.docx]

**Supplementary Information**

Inaccuracies of Deterministic Finite-Element Models of Human Middle Ear Revealed by Stochastic Modelling

Table S1 - Range of the uncertain parameters used in stochastic FE model. CV is defined as the percentage of the standard deviation divided by the mean.

| **Parameter** | **Range for CV of 10%**  **[min,max]** | **Range for CV of 20%**  **[min,max]** | **Parameter** | **Range for CV of 10%**  **[min,max]** | **Range for CV of 20%**  **[min,max]** |
| --- | --- | --- | --- | --- | --- |
| Poisson's ratio of TM | [0.31,0.49] | [0.14,0.49] | Young’s modulus of AML (MPa) | [1.26,2.90] | [0.51,3.80] |
| Poisson's ratio of ossicles | [0.21,0.41] | [0.11,0.48] | Young’s modulus of LML (MPa) | [1.35,2.62] | [0.69,3.24] |
| Poisson's ratio of IMJ | [0.28,0.49] | [0.07,0.49] | Young’s modulus of manubrial fold (MPa) | [0.81,1.64] | [0.42,2.08] |
| Poisson's ratio of ISJ | [0.30,0.49] | [0.11,0.49] | Young’s modulus of SML (MPa) | [3.04,6.45] | [1.18,7.99] |
| Poisson's ratio of SAL | [0.32,0.49] | [0.15,0.49] | Thickness of TM (µm) | [50.99,98.68] | [27.98,123.35] |
| Poisson's ratio of PIL | [0.32,0.49] | [0.16,0.49] | Cochlear load (N.s/m) | [0.16,0.36] | [0.08,0.44] |
| Poisson's ratio of AML | [0.34,0.49] | [0.19,0.49] | Damping (α_2_ coefficient) of TM (µs) | [2.50,5.40] | [1.00,6.81] |
| Poisson's ratio of LML | [0.30,0.49] | [0.12,0.49] | Damping (α_2_ coefficient) of ossicles (µs) | [0.26,0.57] | [0.12,0.74] |
| Poisson's ratio of manubrial fold | [0.26,0.49] | [0.02,0.49] | Damping (α_2_ coefficient) of IMJ (µs) | [85.62,171.57] | [41.24,213.14] |
| Poisson's ratio of SML | [0.28,0.49] | [0.08,0.49] | Damping (α_2_ coefficient) of ISJ (µs) | [88.21,170.73] | [46.42,211.45] |
| Young’s modulus of TM (MPa) | [8.19,16.61] | [4.39,21.23] | Damping (α_2_ coefficient) of SAL (µs) | [92.40,168.56] | [54.79,207.11] |
| Young’s modulus of ossicles (MPa) | [9565.60,18522.78] | [5131.20,23045.55] | Damping (α_2_ coefficient) of PIL (µs) | [89.35,171.26] | [48.70,212.52] |
| Young’s modulus of IMJ (MPa) | [21.19,39.97] | [12.37,49.94] | Damping (α_2_ coefficient) of AML (µs) | [87.23,176.81] | [44.46,223.62] |
| Young’s modulus of ISJ (MPa) | [19.32,39.76] | [8.65,49.51] | Damping (α_2_ coefficient) of LML (µs) | [88.07,167.92] | [46.14,205.85] |
| Young’s modulus of SAL (MPa) | [0.94,1.88] | [0.49,2.36] | Damping (α_2_ coefficient) of manubrial fold (µs) | [2.42,5.35] | [0.84,6.70] |
| Young’s modulus of PIL | [1.28,2.60] | [0.56,3.20] | Damping (α_2_ coefficient) of SML (µs) | [86.03,175.57] | [42.07,221.14] |


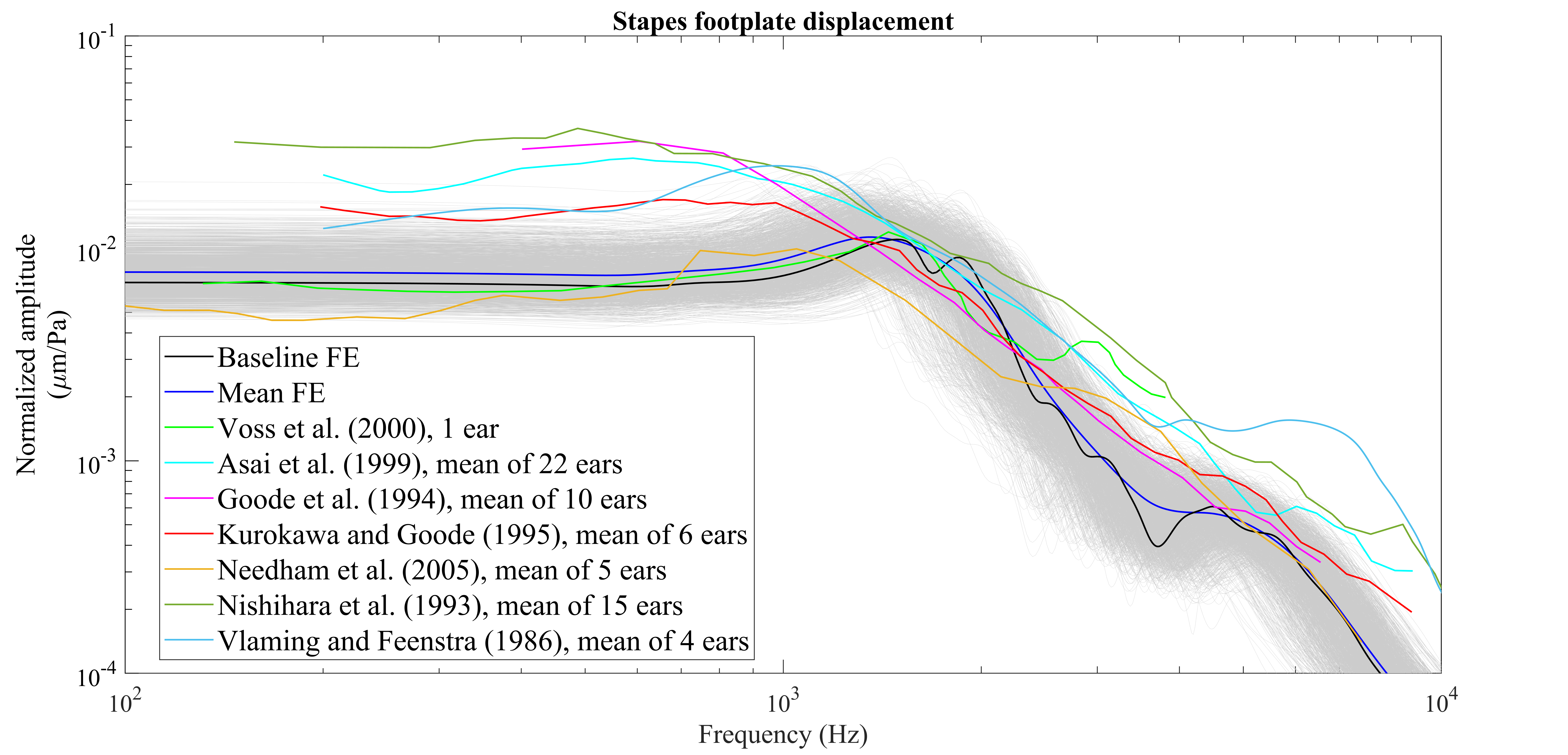


*Figure S1–* ***Comparison of the stochastic FE model results of the stapes footplate with several experimental results in the literature***^1–7^***.*** *The thin gray lines represent the stochastic FE model results of the stapes footplate with CV of 20% for model parameters. All experimental measurement results are reported in the piston-like direction of the stapes footplate. For the works that did not report a value of the measurement angle between the laser beam and the piston-like motion of the stapes, we assumed an angle of 35°. Also, for the works that reported a range for measurement angle between the laser beam and piston-like direction, the mean value of the reported range was used. The experimental results presented in this figure reveal that the variations of the experimental measurements are higher than the maximum variation we considered in our model (CV of 20% for model parameters). The high variation of the experimental results can be due to the morphological variability as well.*

# References

1. Voss, S. E., Rosowski, J. J., Merchant, S. N. & Peake, W. T. Acoustic responses of the human middle ear. *Hear. Res.* **150**, 43–69 (2000).

2. Asai, M., Huber, A. M. & Goode, R. L. Analysis of the best site on the stapes footplate for ossicular chain reconstruction. *Acta Otolaryngol. (Stockh.)* **119**, 356–361 (1999).

3. Goode, R. L., Killion, M., Nakamura, K. & Nishihara, S. New knowledge about the function of the human middle ear: development of an improved analog model. *Am. J. Otol.* **15**, 145–154 (1994).

4. Kurokawa, H. & Goode, R. L. Sound pressure gain produced by the human middle ear. *Otolaryngol. Neck Surg.* **113**, 349–355 (1995).

5. Needham, A., Jiang, D., Bibas, A., Jeronimidis, G. & O’Connor, A. F. The effects of mass loading the ossicles with a floating mass transducer on middle ear transfer function. *Otol. Neurotol.* **26**, 218–224 (2005).

6. Nishihara, S., Aritomo, H. & Goode, R. L. Effect of Changes in Mass on Middle Ear Function. *Otolaryngol. Neck Surg.* **109**, 899–910 (1993).

7. Vlaming, M. & Feenstra, L. Studies on the mechanics of the normal human middle ear. *Clin. Otolaryngol. Allied Sci.* **11**, 353–363 (1986).
